# Supplementary material for: Changes in Cancer Care for Patients Aged 80 and Above: A Cohort Study from Samsung Comprehensive Cancer Center in South Korea
Source: Cancers (Basel). 2025 Jun 17;17(12):2017. doi: 10.3390/cancers17122017 (PMC12191012; doi:10.3390/cancers17122017)
Supplement: Supplementary file 1 [file cancers-17-02017-s001.zip › cancers-3669097-Supplementary Table.pdf]

Supplementary Table S1. Baseline characteristic by cancer treatment among patients aged ≥80 years

Characteristics	Untreated N = 5,867	Treated N = 7,244	P value
**Age at diagnosis (years), mean (SD)**	83.4 (3.3)	82.9 (2.9)	<0.01
**Sex, male**	3,593 (61.2%)	4,325 (59.7%)	<0.01
**Body mass index (kg/m^2)**			
Underweight (≤18.5 kg/m^2)	410 (7.0%)	458 (6.3%)	
Normal (18.5 – 23 kg/m^2)	1,682 (28.7%)	2,615 (36.1%)	
Overweight (23 – 25 kg/m^2)	835 (14.2%)	1,706 (23.6%)	
Obese (> 25 kg/m^2)	842 (14.4%)	1,983 (27.4%)	
Unknown	2,098 (35.8%)	482 (6.7%)	
**Residence area**			<0.01
Seoul	1,579 (26.9%)	2,092 (28.9%)	
Others	4,004 (68.2%)	4,922 (67.9%)	
Unknown	284 (4.8%)	230 (3.2%)	
**Marital status at diagnosis, married**			<0.01
Unmarried	673 (11.5%)	1,424 (19.7%)	
Married	1,927 (32.8%)	4,938 (68.2%)	
Unknown	3,267 (55.7%)	882 (12.2%)	
**Working status at diagnosis**			<0.01
White color	173 (2.9%)	417 (5.8%)	
Blue color	290 (4.9%)	541 (7.5%)	
Service	9 (0.2%)	25 (0.3%)	
Others	8 (0.1%)	13 (0.2%)	
No work	5,322 (90.7%)	6,067 (83.8%)	
Unknown	65 (1.1%)	181 (2.5%)	
**Type of cancer**			<0.01
Lip, oral cavity and pharynx	86 (1.5%)	113 (1.6%)	
Esophagus	121 (2.1%)	176 (2.4%)	
Stomach	907 (15.5%)	1,104 (15.2%)	
Colon and rectum	657 (11.2%)	1,156 (16.0%)	
Liver	421 (7.2%)	377 (5.2%)	
Gallbladder etc.	298 (5.1%)	330 (4.6%)	
Pancreas	373 (6.4%)	267 (3.7%)	
Larynx	33 (0.6%)	44 (0.6%)	
Lung	1,124 (19.2%)	1,359 (18.8%)	
Breast	66 (1.1%)	178 (2.5%)	
Cervix uteri	54 (0.9%)	54 (0.7%)	
Corpus uteri	10 (0.2%)	38 (0.5%)	
Ovary	19 (0.3%)	47 (0.6%)	
Prostate	486 (8.3%)	570 (7.9%)	
Testis	1 (0.0)	0 (0.0%)	
Kidney	84 (1.4%)	133 (1.8%)	
Bladder	201 (3.4%)	227 (3.1%)	
Brain and central nervous system (CNS)	73 (1.2%)	48 (0.7%)	
Thyroid	81 (1.4%)	50 (0.7%)	
Hodgkin lymphoma	1 (0%)	6 (0.1%)	
Non-Hodgkin lymphoma	125 (2.1%)	200 (2.8%)	
Multiple myeloma	38 (0.6%)	66 (0.9%)	
Leukemia	43 (0.7%)	42 (0.6%)	
Other and unspecified	565 (9.6%)	659 (9.1%)	
**SEER stage**			<0.01
Localized	1,293 (22.0%)	2,876 (39.7%)	
Regional	1,435 (24.5%)	2,764 (38.2%)	
Distant	1,684 (28.7%)	1,470 (20.3%)	
Unknown	1,455 (24.8%)	134 (1.8%)	
