# Supplementary figures and images for: Changes in Cancer Care for Patients Aged 80 and Above: A Cohort Study from Samsung Comprehensive Cancer Center in South Korea
Source: Cancers (Basel). 2025 Jun 17;17(12):2017. doi: 10.3390/cancers17122017 (PMC12191012; doi:10.3390/cancers17122017)

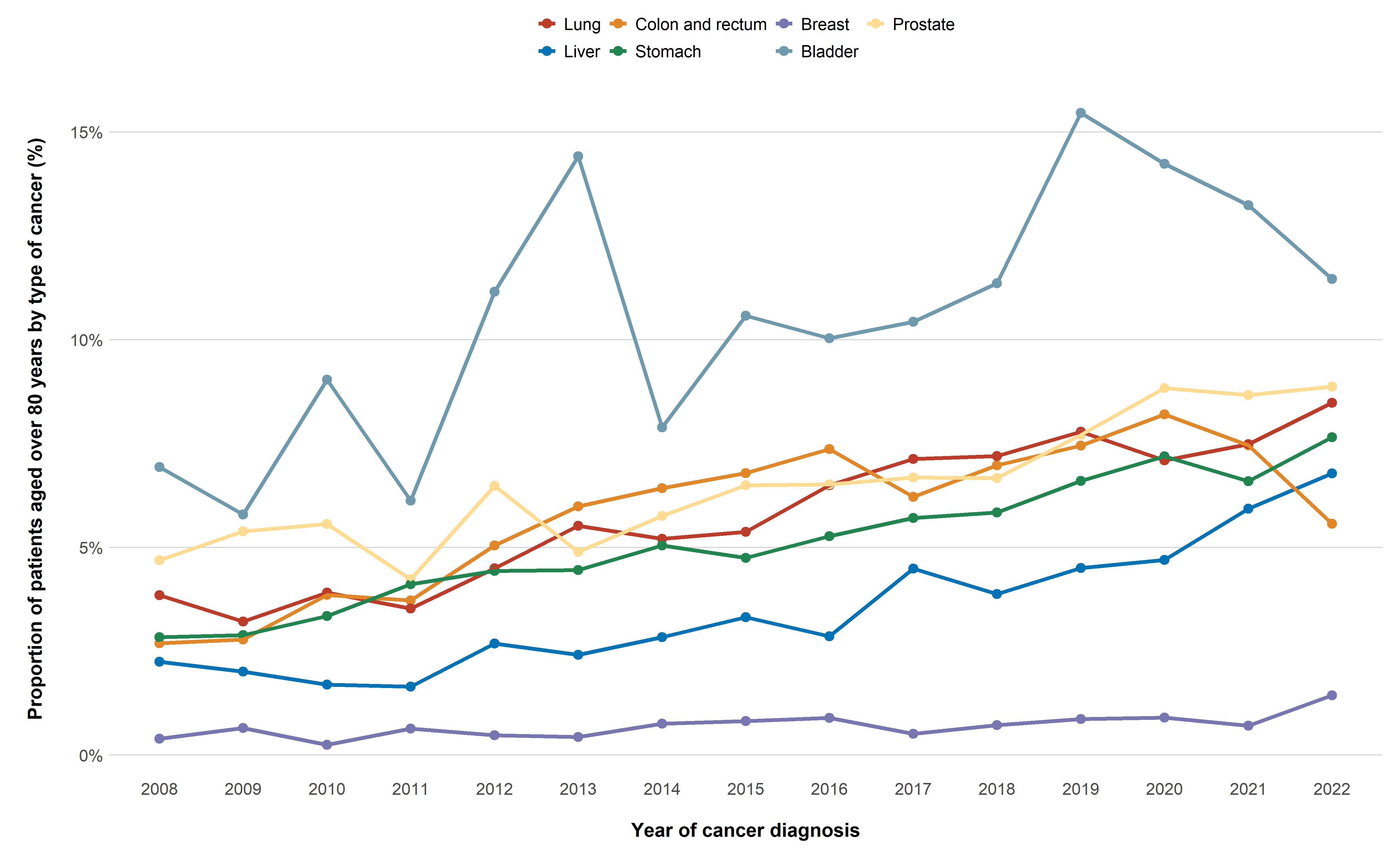

Supplement: Supplementary file 1 [file cancers-17-02017-s001.zip › cancers-3669097-Supplementary Figure.tiff]
